# Supplementary material for: Integrase-mediated differentiation circuits improve evolutionary stability of burdensome and toxic functions in E. coli
Source: Nat Commun. 2022 Nov 10;13:6822. doi: 10.1038/s41467-022-34361-y (PMC9649629; doi:10.1038/s41467-022-34361-y)
Supplement: Supplementary file 3 — Description of Additional Supplementary Files [file 41467_2022_34361_MOESM3_ESM.pdf]

Description of Additional Supplementary Files

File Name: Supplementary Data 1
Description: This excel file contains the sequences of genomic integrations for 1x naïve (eRWnaive1X), 2x naïve (eRWnaive2X), 1x differentiation (eRWdiff1X), and 2x differentiation (eRWdiff2X) at the P21 (T), HK022 (H), Φ186 primary (O1), and Φ186 secondary (O2) integration sites.

File Name: Supplementary Data 2
Description: This excel file contains the full analysis of the sequencing data described in the text and Supplementary Figure 16. Analysis is organized by strain (naive1x, naive2x, diff1x, diff2x, termdiff1x, termdiff2x) and locus sequenced: ColE1 plasmid, P21 (T), HK022 (H), Φ186 primary (O1), and Φ186 secondary (O2) integration sites.

File Name: Supplementary Data 3
Description: This excel file contains the names and sequences of primers used.
