# Supplementary material for: Integrase-mediated differentiation circuits improve evolutionary stability of burdensome and toxic functions in E. coli
Source: Nat Commun. 2022 Nov 10;13:6822. doi: 10.1038/s41467-022-34361-y (PMC9649629; doi:10.1038/s41467-022-34361-y)
Supplement: Supplementary file 7 — Reporting Summary [file 41467_2022_34361_MOESM7_ESM.pdf]

nature portfolio

Corresponding author(s): Rory L. Williams

Last updated by author(s): Nov 3, 2022

Reporting Summary

Nature Portfolio wishes to improve the reproducibility of the work that we publish. This form provides structure for consistency and transparency in reporting. For further information on Nature Portfolio policies, see our Editorial Policies and the Editorial Policy Checklist.

Please do not complete any field with "not applicable" or n/a. Refer to the help text for what text to use if an item is not relevant to your study. For final submission: please carefully check your responses for accuracy; you will not be able to make changes later.

Statistics

For all statistical analyses, confirm that the following items are present in the figure legend, table legend, main text, or Methods section.

n/a	Confirmed	
☐	☒	The exact sample size (*n*) for each experimental group/condition, given as a discrete number and unit of measurement
☐	☒	A statement on whether measurements were taken from distinct samples or whether the same sample was measured repeatedly
☒	☐	The statistical test(s) used AND whether they are one- or two-sided<br>*Only common tests should be described solely by name; describe more complex techniques in the Methods section.*
☒	☐	A description of all covariates tested
☒	☐	A description of any assumptions or corrections, such as tests of normality and adjustment for multiple comparisons
☐	☒	A full description of the statistical parameters including central tendency (e.g. means) or other basic estimates (e.g. regression coefficient) AND variation (e.g. standard deviation) or associated estimates of uncertainty (e.g. confidence intervals)
☒	☐	For null hypothesis testing, the test statistic (e.g. *F*, *t*, *r*) with confidence intervals, effect sizes, degrees of freedom and *P* value noted<br>*Give P values as exact values whenever suitable.*
☒	☐	For Bayesian analysis, information on the choice of priors and Markov chain Monte Carlo settings
☒	☐	For hierarchical and complex designs, identification of the appropriate level for tests and full reporting of outcomes
☒	☐	Estimates of effect sizes (e.g. Cohen's *d*, Pearson's *r*), indicating how they were calculated

Our web collection on statistics for biologists contains articles on many of the points above.

Software and code

Policy information about availability of computer code

Data collection

Plate reader data was collected on a Biotek Synergy H1 plate reader using Gen5 software version 3.10.06, except where noted below. Colony counts where reported were counted manually. Flow cytometry data reported in Supplementary Fig. 2 was collected on a Miltenyi Biotech MACSQuant VYB Flow Cytometer equipped with Violet 405 nm, Blue 488 nm, and Yellow 561 nm lasers. Flow Cytometry data shown in Supplementary Fig. 10 was collected on an Attune NxT Acoustic Focusing Cytometer with 96 well autosampler and BL1-BL3, and YL1-YL4 channels. Plate reader data shown in Supplementary Fig. 10 was collected on a Tecan Infinite 200Pro plate reader using Tecan i-control (2.0.10.0). Simulations were run with custom Python code using a manual ODE solver, with stochastic mutations modeled by drawing from a binomial distribution. Code for generating models and running simulations is included in the Source Data and is available on GitHub (https://github.com/rlwillia/terminal-differentiation-for-evolutionary-stability or https://doi.org/10.5281/zenodo.7213995). Plates for colony counts used to generate Supplementary Fig. 16B were imaged on a ChemiDoc MP imager for GFP fluorescence (Alexa 488 channel, 532/28 Filter, 0.04s exposure), mScarlet fluorescence (Alexa 546 channel, 602/50 Filter, 0.3s exposure), and for visualization of all colonies (UV Trans Illumination, 590/110 Filter. 0.488s exposure). Strains with genomic integrations (eRWnaive1X, eRWnaive2X, eRWdiff1X, eRWdiff2X) were whole genome sequenced with a MinION Flow Cell using the Rapid Barcoding Kit (Nanopore SQK-RBK004). Sequencing of amplicons as described in Supplementary Figure 16 was performed on pooled barcoded amplicons prepared according to the manufactures protocol with LSK110 using a Flongle (Nanopore FLO-FLG001) flow cell, MinKNOW 5.1.0, Guppy 6.1.5, and superaccuracy basecalling.

Data analysis

Plate reader data exported from Gen5 software (for Biotek) and Tecan i-control (for Tecan) was converted to tidy dataframes as csv files with custom Python code (Python version 3.7.12, numpy version 1.20.3, pandas version 0.24.2, matplotlib version 3.5.2, seaborn version 0.11.2). Tidy csv files were loaded into Python Jupyter notebooks using pandas for analysis. Data was plotted from pandas DataFrames using Matplotlib and Seaborn, and figures arranged using Affinity Designer. Data from simulations was similarly analyzed and plotted. Flow cytometry data shown in Supplementary Fig. 2 was gated using custom Python code (adapted from https://github.com/andyhalleran/flow_tools) as described below. Flow cytometry data from Supplementary Fig. 10 was analyzed using FlowJo (10.8.1), and csv files analyzed and plotted in Python Jupyter notebooks similarly. For Supplementary Fig. 10B, multichannel RGB images were created in Image Lab (version 6.1) with equivalently transformed images (Alexa 488: high 30000, low 0; Alexa 546: high 30000, low 0; UV Trans: high 60000, low 30000, inverted), and colonies manually counted using an application (COUNT THINGS) on an iPad pro. For analysis of the whole genome sequencing

used to verify the strains (eRWnaive1X, eRWnaive2X, eRWdiff1X, eRWdiff2X), Flye (version 2.8.3) was used for denovo assembly of the genomes. Resulting genomes were annotated in Geneious (Geneious Prime 2021.1) with features from the genomic integrations in ensure only the intended genomic integrations were present, and loci with genomic integrations aligned to reference sequences to ensure the accuracy of the integrations. For the sequencing described in Supplementary Fig. 16, sequences were demultiplexed and analyzed using Maple (https://github.com/gordonrix/maple) using expected reference sequences (WT amplicons for ColE1, naïve T and H integrations, and primary and secondary O integrations; and WT, and recombined (correctly excised, as well as inverted) sequences for T and H differentiation integrations. As this pipeline failed a subset of differentiation cassettes with unexpected deletion/recombination mutations, basecalled reads were annotated with barcode primer sequences, extracted with in silico PCR in Geneious (Geneious Prime 2022.1), and exported as fastq.gz files for de novo assembly using Flye 2.9 (https://github.com/fenderglass/Flye). This de novo assembly was used to analyze all T and H locus amplicons for differentiation/terminal differentiation by alignment to WT, excised, and inverted reference sequences.

For manuscripts utilizing custom algorithms or software that are central to the research but not yet described in published literature, software must be made available to editors and reviewers. We strongly encourage code deposition in a community repository (e.g. GitHub). See the Nature Portfolio guidelines for submitting code & software for further information.

Data

Policy information about availability of data

All manuscripts must include a data availability statement. This statement should provide the following information, where applicable:

- Accession codes, unique identifiers, or web links for publicly available datasets
- A description of any restrictions on data availability
- For clinical datasets or third party data, please ensure that the statement adheres to our policy

Source data for all main text and supplementary figures are available in the Source Data file. The source data and Python Jupyter notebooks used for analysis and plot generation are also available on GitHub (https://github.com/rlwillia/terminal-differentiation-for-evolutionary-stability). The sequences of all plasmids have been deposited in Genbank (pRW01-13 as OP654158-OP654170). The de novo assembled genomic sequences of strains eRWnaive1X (SAMN31276766), eRWnaive2X (SAMN31276767), eRWdiff1X (SAMN31276768), and eRWdiff2X (SAMN31276769) are avaible on Genbank, and the sequences of each genomic integration is available in Supplementary Data 1. The sequencing data described in the text and Supplementary Figure 16 is available in Supplementary Data 2, and primer sequences available in Supplementary Data 3.

Field-specific reporting

Please select the one below that is the best fit for your research. If you are not sure, read the appropriate sections before making your selection.

☒ Life sciences ☐ Behavioural & social sciences ☐ Ecological, evolutionary & environmental sciences

For a reference copy of the document with all sections, see nature.com/documents/nr-reporting-summary-flat.pdf

Life sciences study design

All studies must disclose on these points even when the disclosure is negative.

Sample size	Experiments characterizing the performance of circuit components and for evaluating the expression of functional dnaseI (Figure 4; Supplementary Figures 2-4, 6-12, 27, 29-30) were done in biological triplicate (with the exception of the preliminary experiment in Supplementary Figure 2F which was in biological duplicate), with replicates showing good reproducibility. Long duration evolution experiments (Figure 2, 3F, Supplementary Figures 13-15, Supplementary Figure 28) were performed with 8 biological replicates. No sample size calculations were performed, though the authors expected more variability between biological replicates with the longer duration experiments than shorter experiments due to stochastic effects, namely mutations. The total number of samples was limited by the capacity of the shaking incubator used, and 8 biological replicates was the maximum possible that allowed all desired experimental conditions to be tested. As seen in Figure 2, 3F, and Supplementary Figure 28, 8 biological replicates allows both the mean and degree of variability between replicates to be observed.
Data exclusions	No data were excluded. However, in the plating and sequencing experiment described in Supplementary Figure 16, high quality sequences could not be obtained for a small subset (diff2X-1 H locus, diff2X-2 H locus, diff2X-3 H locus, diff1X-3 T locus, diff2X-3 T locus) due to either failed PCR or insufficient number of reads.
Replication	Experiments were performed in biological triplicate (Figure 4; Supplementary Figures 2-4, 6-12, 27, 29-30) with the exception of the preliminary experiment in Supplementary Figure 2F which was in biological duplicate due to 96 well plate constraints) or with 8 biological replicates (Figure 2, 3F, Supplementary Figures 13-15, 28). Biological replicates are from isolated colonies picked after transformation of E. coli strains with plasmid(s). Experiments for validating the function of circuit components (Supplementary Figures 3-4, 12, 27, 29) were conducted in a similar manner at least twice, and supported by other preliminary characterization. The experimental conditions for the experiments performed in Figure 2, 3F, and Supplementary Figure 28 were informed by the experiments shown in Supplementary Figures 6-9 and 12. The long duration experiments described in Figure 2, Figure 3F and Supplementary Figure 28 were conducted several times with variations in the duration of the experiment (number of plate generations), and in the number of replicates, experimental conditions (inducer concentration combinations tested). These replications are in agreement with the data reported in the paper. The experiments shown in Figure 2, 3F, Supplementary Figures 13-15, and Supplementary Figure 28 (KanR and AmpR ColE1 versions of the 16 plate generation evolution experiment) were performed at the same time with the same media and reagents (with the exception of relevant antibiotics kanamycin and carbenicillin) to allow for direct comparison. The experiment shown in Supplementary Figure 10 was conducted in order to assess the fraction of differentiated cells as a function of inducer concentration. This specific experiment with flow cytometry analysis was conducted only once in response to a reviewer comment, however the experimental conditions largely overlapped with those from the experiment described in Figure 2, and the plate reader results obtained are in agreement. For dnaseI expression experiments, transformations of the insulated dnaseI

plasmid and control GFP plasmid were performed twice, with the second replication that is reported (Supplementary Table 2) performed for accurate colony counting. Assessing the expression of dnaseI (Figure 4, Supplementary Figure 30) was performed in biological triplicate in parallel, with each biological replicate assayed in technical triplicate.

Randomization	For all experiments, biological replicates were from independent colonies resulting from the transformation of E. coli strains with plasmid(s). Single colonies were randomly picked, outgrown in media with the appropriate antibiotics and inducers (Las AHL for 1x/2x differentiation strains), then culture from each randomly picked colony was diluted into all experimental conditions being tested. All replicates were included in the analysis.
Blinding	The investigators were not blinded to the layout of experiments. This information was required to properly set up the experiment with each strain grown in the correct set of conditions. Data was collected automatically in 96 well or 384 well format by a plate reader or with a flow cytometer with 96 well autosampler. The layout of each experimental plate was documented in a metadata csv file, and analysis and plotting done in Python without any manual manipulation of data from individual samples

Reporting for specific materials, systems and methods

We require information from authors about some types of materials, experimental systems and methods used in many studies. Here, indicate whether each material, system or method listed is relevant to your study. If you are not sure if a list item applies to your research, read the appropriate section before selecting a response.

Materials & experimental systems

n/a	Involved in the study
☒	☐ Antibodies
☒	☐ Eukaryotic cell lines
☒	☐ Palaeontology and archaeology
☒	☐ Animals and other organisms
☒	☐ Human research participants
☒	☐ Clinical data
☒	☐ Dual use research of concern

Methods

n/a	Involved in the study
☒	☐ ChIP-seq
☐	☒ Flow cytometry
☒	☐ MRI-based neuroimaging

Flow Cytometry

Plots

Confirm that:

☒ The axis labels state the marker and fluorochrome used (e.g. CD4-FITC).

☒ The axis scales are clearly visible. Include numbers along axes only for bottom left plot of group (a 'group' is an analysis of identical markers).

☒ All plots are contour plots with outliers or pseudocolor plots.

☒ A numerical value for number of cells or percentage (with statistics) is provided.

Methodology

Sample preparation	Flow cytometry was used in Supplementary Figure 2 and Supplementary Figure 10-11. For Supplementary Figure 2, immediately after the conclusion of growth, cells were diluted 1:50 into 0.1mL PBS in 96 well plates and kept on ice before analysis with flow cytometry. For Supplementary Figure 10, immediately after the conclusion of plate reader growths, cells were diluted 1:100 into 0.9% NaCl.
Instrument	For Supplementary Figure 2, a Miltenyi Biotech MACSQuant VYB Flow Cytometer equipped with Violet 405 nm, Blue 488 nm, and Yellow 561 nm lasers. sfGFP was measured using the 405 nm laser with 525/50 nm filter, and mScarletI was measured using the 561 nm laser with 661/20 nm filter. For Supplementary Figure 10, an Attune NxT Acoustic Focusing Cytometer wth an attached autosampler, and BL1(GFP) and YL1(mScarlet) channels used.
Software	For Supplementary Figure 2, the Miltenyi Biotech MACSQuant VYB Flow Cytometer had a built in computer interface with MACSQuantify™ software. FCS files were converted to tidy csv files using fcsparser (version 0.2.0). Custom Python code for gating and plotting was modified from publicly available repository: https://github.com/andyhalleran/flow_tools. For Supplementary Figure 10, Attune Nxt Software v3.1.2 was used for acquisition, and FlowJo (10.8.1) used for gating and analysis.
Cell population abundance	No cell-sorting/FACS was used in this study.
Gating strategy	For Figure S2, 50,000 ungated events were recorded, and cells were gated on FSC-A and SSC-A, with cells between the 10th and 90th percentile of both being carried through. Peak locations for sfGFP and mScarlet were determined from KDE fits of ungated flow data, gaussian mixture models used to assign cells to peaks, and cells within peaks were designated positive or negative for the respective fluorescent protein using a chosen threshold for peak mean. Peaks with mean log10(mScarletI) >3 were designated as differentiated in Supplementary Figure 2F. For Supplementary Figure 10, populations were first gated on FSC-H and SSC-H to isolate the cell population (>10,000 cells per sample), and BL1-H and YL1-H channels used to characterize

GFP and mScarlet fluorescence, respectively. Samples with known populations of GFP +/- and RFP +/- were used to determine gating. GFP+ fraction reported in reported in Figure S10C.

☒ Tick this box to confirm that a figure exemplifying the gating strategy is provided in the Supplementary Information.

This checklist template is licensed under a Creative Commons Attribution 4.0 International License, which permits use, sharing, adaptation, distribution and reproduction in any medium or format, as long as you give appropriate credit to the original author(s) and the source, provide a link to the Creative Commons license, and indicate if changes were made. The images or other third party material in this article are included in the article's Creative Commons license, unless indicated otherwise in a credit line to the material. If material is not included in the article's Creative Commons license and your intended use is not permitted by statutory regulation or exceeds the permitted use, you will need to obtain permission directly from the copyright holder. To view a copy of this license, visit http://creativecommons.org/licenses/by/4.0/
